# Mpox: A case study for a one health approach to infectious disease prevention

**DOI:** 10.1016/j.onehlt.2025.101059

**Published:** 2025-05-02

**Authors:** David T.S. Hayman, Marion P.G. Koopmans, Andrew A. Cunningham, Salome A. Bukachi, Leandre Murhula Masirika, Wanda Markotter, Thomas C. Mettenleiter

**Affiliations:** a Molecular Epidemiology and Public Health Laboratory, Hopkirk Research Institute, Massey University, Palmerston North, New Zealand; b Erasmus MC, Department of Viroscience, Rotterdam, the Netherlands; c Institute of Zoology, Zoological Society of London, London, UK; d Institute of Anthropology, Gender and African Studies, University of Nairobi, Nairobi, Kenya; e Department of Anthropology, Durham University, Durham, UK; f Centre de Recherche en Sciences Naturelles de Lwiro, South -Kivu, DS Bukavu, Democratic Republic of the Congo; g Congo Outbreaks, Research and Development, South-Kivu, Bukavu, Democratic Republic of the Congo; h SaBio Instituto de Investigación en Recursos Cinegéticos IREC (Universidad de Castilla-La Mancha & CSIC), Ronda de Toledo 12, Ciudad Real, Spain; i Center for Viral Zoonoses, Department of Medical Virology, Faculty of Health Sciences, University of Pretoria, Pretoria, South Africa; j Friedrich-Loeffler-Institut, Federal Research Institute for Animal Health, Südufer 10, Greifswald - Insel Riems, Germany

**Keywords:** Monkeypox virus, Zoonotic spillover, Reservoir hosts, Infectious disease emergence, Drivers, Epidemiology

## Abstract

Mpox has been declared a global health emergency twice by the World Health Organization due to its impacts within and beyond Africa. Enzootic in Central and West African wildlife, mpox outbreaks have resulted from zoonotic spillover, with recent events revealing increased human-to-human transmission. Factors like population growth and environmental disruption, alongside reduced smallpox immunity, increase emergence risk. In addition, the emergence in South Kivu of a distinct subclade of mpox virus points at a currently understudied aspect of mpox virus lineages and their dynamics in reservoir hosts. A One Health approach—integrating human, animal, and environmental science—is essential for reducing the risk of mpox emergence. This approach should encompass ecological studies to understand putative reservoir population dynamics and the potential for interventions, reducing activities that increase human-animal contacts, respectful community engagement to reduce spillover risk from cultural practices (such as hunting multiple species of wildlife for consumption), and socially acceptable and equitable access to medical and non-medical countermeasures to prevent or control ongoing human-to-human transmission. Politically supported collaborative efforts across disciplines with involvement of stakeholders are critical to promote and strengthen socially and environmentally sustainable practices to mitigate future outbreaks.

## Highlights

• Human-human spread and zoonotic spillover of mpox outbreaks is worsened by population growth, habitat change and low immunity
• A distinct mpox virus subclade in South Kivu, DRC, highlights the need for further research on virus lineages and reservoir host dynamics.
• A One Health approach is key to prevention, using ecology, engagement, and equitable access to interventions.

## 1 Introduction

On 14 August 2024, the World Health Organization declared mpox a global public health emergency of international concern (PHEIC) for the second time in just over two years [1]. Mpox, formerly known as monkeypox, is a zoonotic viral disease that has been a significant public health concern in Africa for decades [2].

Human disease, caused by various monkeypox virus (MPXV) clades, was first identified in the Democratic Republic of Congo (DRC) in 1970 [3]. Until recently, it has predominantly affected people in Central and West African countries, where the viruses remain enzootic in wildlife reservoirs with sporadic local epidemics [2]. Despite being overshadowed by other infectious diseases, like Ebola virus disease (EVD) and HIV/AIDS, mpox has persisted as a health challenge, evolving in both its epidemiology and global impact [4,5].

In recent years, mpox has gained global attention due to its emergence outside Africa, with significant epidemics of clade IIb reported in Europe, North America, and Asia in 2022 [4,[6], [7], [8], [9]]. In 2017, Nigeria experienced its largest epidemic in nearly four decades, which marked a turning point in the understanding of mpox epidemiology [10]. In 2022 a global outbreak was triggered by a clade IIb virus entering into networks of men who have sex with men in the UK and Europe [[11], [12], [13]]. Research in response to this outbreak uncovered evidence for undetected person-to-person transmission in Africa, in addition to sporadic outbreaks due to zoonotic spillover [14].

Here we review historical and current events and highlight evidence why a One Health approach, including primary, deep prevention through reducing the drivers of emergence [15], is key to reducing mpox emergence risk.

## 2 Historical background

The first recognized human cases of mpox were reported in the DRC, formerly Zaire, in the early 1970s [3]. The virus was initially identified in monkeys in a laboratory setting in 1958, which led to the misnomer “monkeypox” [16]. However, there is increasing (albeit limited) evidence that rodents in the African rainforest are the primary reservoir hosts [[17], [18], [19]]. The disease was initially thought to be rare, often presenting with symptoms similar to smallpox, including fever, rash, and swollen lymph nodes [20].

In the decades following its discovery, mpox has remained largely confined to remote areas, with sporadic outbreaks occurring in Central and West Africa [21]. Although often limited in scale, these highlighted the persistent threat of the virus to human populations in areas where people live in proximity to infected wildlife. The infection spilled-over primarily through direct contact with infected animals, especially during hunting or handling wild animals or their products, with onward spread through more limited person-to-person transmission via contact with bodily fluids [3,20], although clear epidemiological data are frequently missing [21,22]. However, the global eradication of smallpox in 1980 [23], achieved through widespread vaccination, indirectly affected mpox. Both smallpox infection and the vaccinia virus-based vaccine used for the elimination campaign provided cross-protection against MPXV. With the cessation of smallpox vaccination following smallpox eradication, the incidence of mpox cases began to rise, particularly in rural and forested regions of tropical Africa where the viruses remain enzootic among wildlife [2,24].

Mpox is caused by the double-stranded DNA *Orthopoxvirus* monkeypox virus (MPXV) [6,7,9]. The virus is genetically and antigenically related to the smallpox-causing variola virus, which before eradication was a leading cause of human deaths and morbidity globally. The diseases are similar, with symptoms including fever, rash, and pustules, although MPXV generally causes milder illness [8] and the virus is less transmissible among people than the highly infectious variola virus [25]. There are two MPXV subtypes, clade I and clade II, sharing ∼99.4 % sequence identity at the protein level and 96.5 % at the nucleotide level [26]. The 2022–2023 pandemic was caused by a clade IIb virus which also circulates in the African region, mostly in West-Africa, whereas clade I is largely limited to Central Africa and has been associated with more severe disease and higher mortality in people than clade II viruses [6,7,9,27]. Evidence for these clade-associated differences in disease severity, however, is limited and factors such as strengthening surveillance including the use of serology can impact metrics such as case-fatality estimates [28,29]. While smallpox was transmitted efficiently between humans both by the respiratory route and through direct contact, transmission of MPXV among humans currently is less effective, except for sexual transmission and transmission in close contacts, notably children in households of cases [30,31]. Therefore, the epidemiology of mpox in the African region is quite complex, with evidence of relatively frequent zoonotic infection from animal reservoirs where the pathogenic viruses circulate naturally, in addition to undetected human to human transmission, which complicates its control [6,7,13,14,21,27,[32], [33], [34]].

The relationship between variola virus (the cause of smallpox) and MPXV, and uncertainty as to whether or not MPXV could evolve to increase transmissibility among people, underscores the importance of surveillance, contact tracing, isolation and quarantine, and vaccination in managing viral outbreaks. However, there also is a need for better prevention of spillover and emergence in the first place. This requires a One Health approach that considers both direct and indirect human interactions with animal reservoirs and the ecosystems within which they live, alongside anthropological, sociological and community health work [15]. MPXV can also affect wildlife [19], with examples including large outbreaks in chimpanzees [35]. Better understanding of MPXV dynamics in the reservoir hosts, including the impact of activities that impact ecosystems and infection prevalence within hosts, and an understanding of the impact of MPXV on (endangered) wildlife species might also contribute to improved wildlife health and conservation outcomes [19]. Such mitigation measures could be simultaneously beneficial for human and wildlife health and for the environment. If these approaches are not taken, zoonotic transmission and subsequent outbreaks will continue.

## 3 The current situation and challenges

**Fig. 1:**
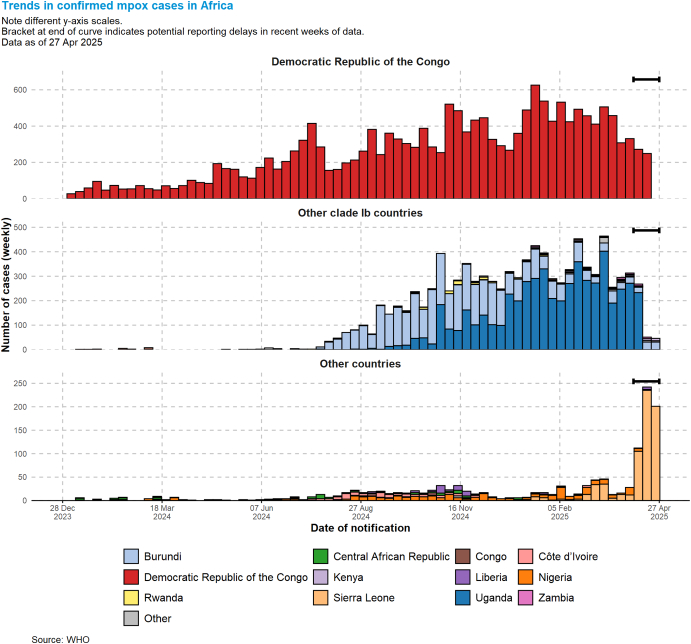
The epidemic curves for MPXV clade 1b by affected countries. Source: https://worldhealthorg.shinyapps.io/mpx_global/. Accessed 28/4/2025.

In 2023, an MPXV spillover triggered an ongoing human-to-human outbreak that also appears to be mostly driven by (hetero)sexual transmission [36], leading to the 2024 PHEIC declaration [32]. Since its initial detection in South Kivu, the virus spread within the region and into neighbouring countries with a few cases in international travellers (https://worldhealthorg.shinyapps.io/mpx_global/) (Fig. 1). In addition, countries like the DRC, Nigeria, and the Central African Republic continue to report the highest numbers of cases in their history [37], and it has become increasingly clear that this is explained by an entangled mix of zoonotic spillovers [7] and person-to-person transmission events including through sexual and non-sexual transmission [31,38,39]. The 2017 Nigerian outbreak showed that mpox could cause severe illness and that human-to-human transmission was likely more efficient than previously assumed [10]. This greatly complicates the current situation in Africa [37].

Well-established challenges for addressing mpox in Africa include underreporting due to under-resourced healthcare, surveillance and reporting systems (meaning the true disease burden is higher than official figures show), and identifying outbreaks in their early stages making the implementation of effective control measures difficult [4,7,9,40,41]. The ongoing 2022 mpox pandemic was not recognized until cases emerged outside Africa (https://www.who.int/emergencies/disease-outbreak-news/item/2022-DON381). These limitations also make the identification of primary cases difficult, limiting epidemiological investigations of spillover events to identify risk factors. Internal and transboundary conflicts also impact surveillance and mitigation measures (https://healthpolicy-watch.news/conflict-in-goma-sets-back-mpox-treatment/). Moreover, recent withdrawal of US support for mpox control programmes also demonstrate the extent to which political decisions by a single country can undermine mitigation strategies (https://healthpolicy-watch.news/from-mpox-to-influenza-usaid-collapse-and-cdc-blackout-upend-who-response-to-deadly-outbreaks/), providing evidence that diversification of donor sources and ultimately moving away from donor-based work will help build resilience [42].

The ecological impacts of land-use changes, such as agricultural expansion, urbanisation and resource extraction like mining and logging, drive the emergence of diseases, including mpox, while ongoing challenges of poverty, conflict, and displacement in parts of Africa complicate efforts to study, control and prevent MPXV emergence and spread [40]. Yet the lack of access to vaccines and treatments in the region are obviously not only due to these challenges. The smallpox vaccine, which offers significant (85 %) protection against mpox [43], is not widely available in Africa, and newer vaccines specifically targeting mpox are in short supply [44,45], compounding global inequities in vaccine distribution. Only in November 2024 did the Access and Allocation Mechanism for mpox allocate an initial 899,000 vaccine doses for the nine African countries most impacted by the current increase in mpox (https://www.who.int/news/item/06-11-2024-vaccine-doses-allocated-to-9-african-countries-hardest-hit-by-mpox-surge). While positive, a key challenge is how to prioritize vaccines in populations where sources of infection that are not always clear and where logistical issues and cultural stigma may prevent risk-targeting vaccination campaigns.

## 4 The importance of one health for the prevention of Mpox

**Fig. 2:**
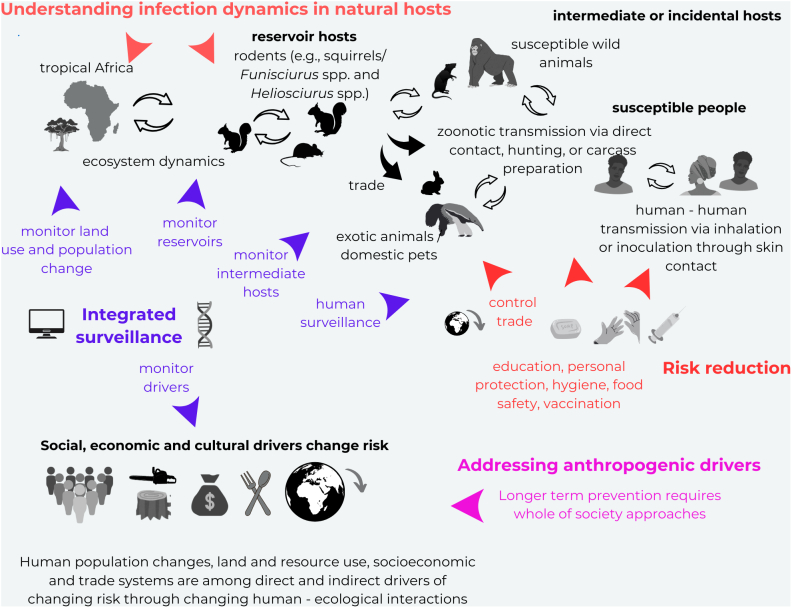
A One Health approach to mpox prevention. The main ecological and epidemiological processes and direct and indirect drivers of infectious disease emergence are shown, with key steps to prevent mpox spillover and outbreaks in people shown in coloured text and coloured by previously published themes [15], with arrows indicating where these take place. Deep, sustainable prevention requires addressing the social, cultural and economic anthropogenic drivers of spillover [15] which are frequently transboundary issues.

The concept of One Health, which recognizes the interconnectedness of human, animal, and environmental (ecosystem) health, is crucial for the prevention and control of mpox spillovers [15,46]. One Health emphasizes the transdisciplinary collaboration between multiple disciplines—ranging from human medicine to veterinary science, ecology, anthropology and public health—to address health threats at the human-animal-environment interface [46], with numerous case studies highlighting successful approaches to aspects of this approach within Africa [[47], [48], [49], [50]]. The interconnectedness of ecosystems, human populations, and animal reservoirs means that addressing the root causes of mpox zoonotic spillover requires a coordinated response that considers all three components (Fig. 2).

Environmental factors play an important role in the emergence and spread of mpox. Data on the natural reservoir hosts is incomplete, but these likely include four widely distributed forest dwelling rodent species in the three African rainforests, the Congo Basin, and the Upper and Lower Guinean forests [17]. Most recently, the first infected fire-footed rope squirrel (*Funisciurus pyrropus*) was reported following an mpox outbreak in wild sooty mangabeys (*Cercocebus atys*) in Côte d'Ivoire, affecting one third of the group and killing four infants in 2023 [19]. To trace the source, researchers screened local wildlife and identified a dead MPXV-infected rope squirrel 3 km from the mangabeys' range 12 weeks earlier, with an identical genome to the mangabey MPXV. Dietary investigations, including video evidence and faecal metabarcoding, confirmed that mangabeys consumed this squirrel species, linking the outbreak to a rare direct interspecies transmission event, detected through long-term health monitoring. This highlights the benefits of long-term ecological studies which include health monitoring.

Deforestation and habitat destruction disrupt ecosystems, frequently leading to increased human-wildlife contact and increased risk of subsequent transmission of zoonotic diseases [51,52]. Identifying hotspots of these activities can help focus surveillance activities and control measures to reduce mpox infection risk [[53], [54], [55]]. Changes in human behaviours and land use, such as changes in species hunted for wild meat and encroachment into forests, can increase the likelihood of exposure of people to wildlife pathogens. Increases in human population density and movement can increase the risk of human-to-human transmission following spillover events [56]. Countries at high risk of mpox emergence, such as DRC, have high rates of deforestation and forest fragmentation due to shifting agriculture [57,58]. Therefore, monitoring changes in human population and land use [51,59], as well as in human behaviours [33,[60], [61], [62]], can improve our understanding of the spillover risks and help to plan mitigation measures [54,63].

Mitigation measures may include reductions of human-animal interactions at the edges of increasingly fragmented forests through a range of community interventions [64], alongside planning to minimise and prevent further forest encroachment and fragmentation, yet in many African communities traditional practices such as hunting and consuming wild animals are deeply rooted in cultural and economic life [65,66]. This requires acknowledging that there are social and cultural connections that must be respected and local knowledge systems that should be included, necessitating integrated policy and governance across multiple institutional scales [[67], [68], [69]]. For example, public health education and community engagement and involvement are critical for preventing the spread of mpox [55]. Public health campaigns that respect socio-cultural and economic logics and practices as well as recognise the indigenous and informal education and knowledge systems while sensitizing and educating communities about the risks of MPXV transmission are essential for reducing the incidence of zoonotic spillover [53,70] (Fig. 2).

The factors that determine disease emergence are also frequently transboundary, both in terms of ecosystem boundaries (e.g., between forests, agricultural systems such as agroforestry and subsistence farming, and urban areas) and national borders. Therefore, regional and broader geographic collaboration is required alongside local actions. Transboundary trade has led to MPXV emergence in the USA [71] and trade is also a driver of land use change [58], though commodity driven deforestation is greater in Southeast Asian and South American tropical forests than in Africa, where shifting agriculture with more temporary crop cultivation for subsistence is the greater driver of deforestation [57,58]. Understanding the drivers of land use change and mitigating these are medium- to long-term goals that could reduce the risk of mpox emergence, as well as that of other diseases such as EVD [51,52]. All mitigating actions likely require some trade-off between immediate needs, such as food [50] and incomes (for workers, private companies and governments), and longer-term risks along with an acknowledgement of who bears the burden of these changes [72]. Yet by promoting environmental stewardship and sustainable practices, One Health can help mitigate these risks, thereby reducing the likelihood of mpox outbreaks.

Based on the above, capacity building programs ideally would include monitoring infection in wild reservoirs (e.g. rodents) and intermediate hosts (e.g. non-human primates) of MPXV [16,17,20]. Surveillance of human activities to quickly identify increases in risk though, for example, changing human behaviours or population distributions, should be included and prioritised. However, by improving surveillance systems in wildlife and domestic animals, including for disease outbreak and unusual mortality events, potential risky changes may be detected before spillover into human populations [54] and this may help identify as yet unconfirmed wildlife reservoirs [17,19] and new subclades like the clade Ib currently spreading from eastern DRC to neighbouring countries (Fig. 1). Wildlife surveillance can be especially difficult to do well, whereas domestic animal disease surveillance is likely easier, but including both has benefits [19,54]. This requires expertise from multiple disciplines and collaboration between veterinary, wildlife, public health and laboratory services at least [48]. In Africa, where human-wildlife interaction is common [73], and where there are significant gaps in veterinary public health infrastructure, diagnostic capacity and access to healthcare, the One Health approach is vital [18,64].

## 5 Equity and vaccine access

While the One Health approach is essential for prevention, it also is based on equity [46], and equity in education, research and data collection, health care and vaccine access is equally important for the effective management of mpox [4,44,74]. Historically (and currently), the distribution of resources, including vaccines, has been uneven, with wealthier nations having greater access to supplies [75]. This disparity is clearly visible in Africa, where despite being the epicentre of mpox outbreaks and bearing the brunt of the disease burden, access to vaccines and other preventive measures has been (and still is) limited [76]. Equity and inclusivity in data collection is also critical to development of equitable and inclusive interventions [77]. Disparities across the gender divide have also been noted to contribute to inequities in surveillance and research [78] and evidence suggests women, children and infants may be more likely to contract mpox disease in DRC [[30], [31]].

Once the current epidemic has been brought under control, consideration should be given to the on-going use of vaccination for people at greatest risk of exposure to wildlife reservoirs to reduce the risk of infection reaching the human population in the first place. Targeted vaccination of wild meat hunters and butchers, for example, would help protect the wider community in the absence of widespread availability of mpox vaccines.

There are considerable practical and sociological issues that must be addressed for vaccination programs to be successful [30,74,[79], [80], [81], [82], [83], [84]], and work is necessary to identify the most effective mpox vaccination strategy in mpox endemic settings [2,41,85,86]. Yet equitable vaccine distribution is not just a moral but also a practical imperative. Ensuring availability of vaccines in regions where mpox is endemic, such as parts of Central and West Africa, is crucial for controlling the disease at its source [2]. International cooperation and investment are needed to bolster local health systems, improve vaccine storage and distribution networks, and support public health education campaigns that can increase vaccine uptake.

Furthermore, equity in healthcare extends beyond vaccine access and immunising frontline workers [83] to include the availability of diagnostic tools, treatments, and the overall strengthening of healthcare infrastructure (Supplemental panel). A One Health approach must advocate for resource allocation that prioritizes the most vulnerable populations, including those in low-income regions, to ensure that prevention and treatment measures are accessible to all, but also not to neglect more primary prevention measures to reduce the risk of spill-over in the first place (Fig. 2).

Mpox presents challenges for public health and healthcare workers, veterinarians, veterinary technicians and ecologists (Fig. 2; Supplemental panel). Thus, it also calls for a One Health workforce from the different disciplines. It is advisable to coordinate and streamline sampling and diagnostic procedures in cooperation between human and veterinary laboratories and professionals including multidisciplinary trained technicians to obtain and analyse samples from animals and humans under appropriate biosafety conditions. This includes the proper use of personal protective equipment and the necessary infrastructure for safe analysis and disposal of samples even in remote conditions (Supplemental panel).

## 6 Lessons from and for other systems

Lessons learned from applying a One Health approach to mpox—such as fostering cross-sectoral collaboration, improving zoonotic pathogen surveillance, and engaging and involving communities in prevention efforts—can inform strategies to prevent the emergence of other infections. Conversely, experiences with these infections, including early detection systems, wildlife monitoring, and risk communication and community engagement and involvement, can enhance the effectiveness of One Health responses to mpox. This includes improved hypothesis generation and research, such as relating to the role of deforestation disease emergence (e.g., [52]), the assessment of socioeconomic, cultural, political and historical aspects of the mpox outbreak to understand the context, the multi-sectoral evaluation of potential interventions to understand trade-offs and synergies among response options, and mechanisms to translate findings into policy to ensure there are balanced, sustainable approaches that maintain or improve human, animal and environmental health.

Each infectious disease has evolutionary, ecological and pathogen features that make it unique. Highly pathogenic avian influenza A (HPAI) viruses typically evolve from low pathogenicity wild type viruses in high population densities of poultry following historic spillover from wild birds in which they circulate [87]. Different orthohantaviruses have different hosts globally, causing mostly haemorrhagic fever with renal syndrome in Eurasia (except Seoul virus, which is worldwide in invasive *Rattus* species); or hantavirus cardiopulmonary syndrome in the Americas [88]. Filoviruses may be widespread in Eurasian bats, yet viruses from *Marburgvirus* and *Ebolavirus* genera cause severe human disease such as EVD if they spillover in Africa [89]. Lassa virus is largely maintained and transmitted to people by *Mastomys natalensis* in West Africa [90], yet closely related viruses in the same murine species in East Africa appear not to cause Lassa virus-like disease outbreaks [91].

All these viruses cause limited to no disease in their natural wildlife hosts (e.g. waterfowl, rodents or bats) and have limited human-to-human transmission capabilities. However, chance events can lead to adaptation and increasing opportunities for contacts with new hosts. Moreover, there are numerous related animal viruses that may be predisposed to infect humans but have not yet had the appropriate opportunity to do so. Opportunities for transmission of these viruses will increase through changes in human populations and behaviours, land use change, agricultural systems [63], anthropogenic movement of wildlife [92] and climate change [93,94]. As people and our domestic animals invade more wild, biodiverse habitats, the risk of disease emergence can increase by increasing risks of spill-over [51,52]. The HPAI H5N1 clade 2.3.4.4b is an example of how an infection that evolved in domestic poultry farming systems in one part of the world can have significant global domestic animal and wildlife health, as well as potential human health, impacts [87]. Increasingly connected, large populations, like high density poultry farming for food or eggs [87] or farmed mink for fur [95], or urbanized humans, can facilitate spread and prolong epidemics [96,97] even for infections with limited human-to-human transmission [98], and make them much harder to control [56].

Just as hospital infection prevention and control and food safety measures are in place to mitigate multiple potential hospital- and food-borne infections, so too can One Health approaches be applied to mitigate infection transmission among and between species [15]. We have known for many years [99] that there are hotspots of infectious disease emergence in humans, particularly in rapidly changing parts of the world, such as those impacted by mpox now and by EVD and Lassa fever recently [51,52,68,90,96,100]. There is increasing evidence that countries are learning to better respond to zoonotic disease events (e.g. [100]), yet there is only sporadic evidence of attempts to address the underlying drivers of spill-over, even in wealthy countries [101]. The science behind this is less well understood, but this does not mean it is not evidence based. Similar uncertainty has been used as an excuse to deal with other important global issues, such as climate change [102,103]. Local interventions require community-level engagement and involvement and adaptation to the local contexts [55,64] which may help mitigate the direct (e.g., land use change and resource extraction) and indirect (e.g., socioeconomic changes) drivers of zoonotic spill-over. Yet many of these drivers are directly or indirectly influenced by decisions and actions by people in the public and private sector in other, often distant, locations. Thus, to apply a One Health approach we need to not only work locally, but understand transboundary, socio-political and economic, cultural and gender and equity issues that influence the local changes that increase the risk of infectious disease emergence as well as call for improvements of more direct measures, such as vaccine equity.

## 7 Summary

Mpox has been a persistent public health issue in Africa for over five decades, with the continent bearing the greatest burden of the disease. While the virus has recently gained global attention, it remains a significant challenge in Africa, where weak healthcare systems, underreporting, and limited access to vaccines complicate efforts to control and prevent outbreaks, and ongoing pressures on ecosystems increase the risk of spillovers. Addressing these challenges requires a coordinated One Health approach that includes improving surveillance and understanding of human and ecological factors driving spillovers, engaging and involving communities in public health efforts to reduce spillover risk, and developing vaccination and other prevention strategies that are equitable and include communities at greatest risk of spillovers in the response efforts. Only through these measures can African countries hope to reduce the impact of mpox, protect their populations from this enduring health threat and reduce the risk of viral emergence elsewhere [4].

## CRediT authorship contribution statement

**David T.S. Hayman:** Writing – original draft, Conceptualization. **Marion P.G. Koopmans:** Writing – review & editing, Conceptualization. **Andrew A. Cunningham:** Writing – review & editing, Conceptualization. **Salome A. Bukachi:** Writing – review & editing, Conceptualization. **Leandre Murhula Masirika:** Writing – review & editing. **Wanda Markotter:** Writing – review & editing, Conceptualization. **Thomas C. Mettenleiter:** Writing – review & editing, Conceptualization.

## Declaration of competing interest

The authors declare the following financial interests/personal relationships which may be considered as potential competing interests:

M Koopmans reports financial support was provided by EDCTP under grant agreement No. 101103059 (GREATLIFE) and from HERA (DURABLE). MK and LM received funding from EDCTP project JUA KIVU (grant number 101195116). If there are other authors, they declare that they have no known competing financial interests or personal relationships that could have appeared to influence the work reported in this paper.

## Data Availability

No data was used for the research described in the article.

## Appendix A Supplementary data

Supplementary material: Sampling techniques and diagnostics in a One Health context Supplementary material
