# Supplementary material for: Mpox: A case study for a one health approach to infectious disease prevention
Source: One Health. 2025 May 2;20:101059. doi: 10.1016/j.onehlt.2025.101059 (PMC12135377; doi:10.1016/j.onehlt.2025.101059)
Supplement: Supplementary file 1 — Supplementary material: Sampling techniques and diagnostics in a One Health context [file mmc1.docx]

**Supplemental panel.** Sampling techniques and diagnostics in a One Health context

Mpox presents challenges for One Health practitioners, including public health and healthcare workers, veterinarians, veterinary technicians and ecologists. The decision to test should be based on both clinal, epidemiological and ethnographic data for people and include veterinary, virological and ecological data for other species. Testing should be offered to anyone who meets the mpox case definition to limit risk of spread and initiate epidemiological investigations.

Mpox clinical signs may resemble other infectious diseases, including zoonoses, and non-infectious diseases, making it challenging to differentiate mpox solely based on clinical presentation. In people with signs, other potential causes of discrete skin lesions or disseminated rashes include varicella zoster virus (chickenpox), measles, scabies, herpes simplex virus, *Treponema pallidum* (syphilis) and other orthopoxviruses [1]. MPXV infection may also be asymptomatic among people [2] and, particularly, subclinical in wildlife reservoirs[1]. Sampling during outbreaks should include multidisciplinary trained technicians and can be challenging when samples from wildlife or other domesticated species are required.

Technicians should be trained to use standard operating procedures for the effective and safe collection, storage and handling of possible MPXV positive specimens, including how to properly use personal protective equipment throughout the sampling process in any remote conditions [3]. This includes consideration of contamination and disposal, as any sample taken with the suspicion of MPXV infection should be considered as possible infective. Veterinarian technicians should be trained to handle animals with suspected infection especially when doing necropsies for sampling. Aerosol generating procedures should be avoided, and vaccination for staff handling samples from humans or high-risk animal samples is recommended.

Recommended sample types for MPXV are surface and/or skin materials, such as exudate swabs and lesion crusts [3], including perhaps from genitalia, and other more invasive sampling may be required, especially for wild species or serology, requiring further training. Care and consideration of psychosocial support for human cases is also needed.

Testing is typically by PCR, though serology [4, 5] to whole genome sequencing [6] are useful. Serology for wildlife can be particularly challenging due to the lack of validated assays [7]. However, serological sampling in wildlife and humans is crucial to understand case fatality rates and subclinical infection, as well as for use in risk factor studies. All tests, however, require good samples. Minimizing handling after collection and establishing quick transport to better equipped laboratories helps maintain sample quality. In remote locations, this can be difficult and require the use of portable refrigeration or coolers. Filter paper cards for DNA stabilization and dried blood spot cards for serology offer possible solutions, allowing DNA and blood samples to dry and remain stable for years in dry conditions [4].

Further resources can be found here:

<https://www.who.int/publications/i/item/WHO-MPX-Surveillance-2024.1>

<https://africacdc.org/download/mpox-surveillance-reporting-protocol-for-african-union-member-states/>

<https://www.who.int/emergencies/outbreak-toolkit/disease-outbreak-toolboxes/mpox-outbreak-toolbox>

[1] D. Forni, R. Cagliani, C. Molteni, M. Clerici, and M. Sironi, "Monkeypox virus: The changing facets of a zoonotic pathogen," *Infection, Genetics and Evolution,* vol. 105, p. 105372, 2022/11/01/ 2022, doi: <https://doi.org/10.1016/j.meegid.2022.105372>.

[2] I. De Baetselier *et al.*, "Retrospective detection of asymptomatic monkeypox virus infections among male sexual health clinic attendees in Belgium," *Nature medicine,* vol. 28, no. 11, pp. 2288-2292, 2022.

[3] M. Altindis, E. Puca, and L. Shapo, "Diagnosis of monkeypox virus – An overview," *Travel Medicine and Infectious Disease,* vol. 50, p. 102459, 2022/11/01/ 2022, doi: <https://doi.org/10.1016/j.tmaid.2022.102459>.

[4] C. J. Waddell *et al.*, "Minimally Invasive Blood Collection for an Mpox Serosurvey among People Experiencing Homelessness," *The Journal of Applied Laboratory Medicine,* p. jfae035, 2024.

[5] E. Kinganda-Lusamaki *et al.*, "Use of Mpox Multiplex Serology in the Identification of Cases and Outbreak Investigations in the Democratic Republic of the Congo (DRC)," *Pathogens,* vol. 12, no. 7, p. 916, 2023. [Online]. Available: <https://www.mdpi.com/2076-0817/12/7/916>.

[6] Á. O’Toole *et al.*, "APOBEC3 deaminase editing in mpox virus as evidence for sustained human transmission since at least 2016," *Science,* vol. 382, no. 6670, pp. 595-600, 2023, doi: doi:10.1126/science.adg8116.

[7] A. T. Gilbert *et al.*, "Deciphering serology to understand the ecology of infectious diseases in wildlife," *EcoHealth,* vol. 10, pp. 298-313, 2013.
